# HLA-DQA1*05:01 and DQA1*05:05 inform choice of anti-tumor necrosis factor and concomitant use of immunomodulators in patients with inflammatory bowel disease

**DOI:** 10.1093/ecco-jcc/jjaf195

**Published:** 2025-12-05

**Authors:** Qian Zhang, Mohammed Sharip, Christopher Roberts, Eathar Shakweh, Miles Parkes, Tariq Ahmad

**Affiliations:** Genomics of Inflammation and Immunity Group, Human Genetics Programme, Wellcome Sanger Institute, Wellcome Genome Campus, Hinxton, United Kingdom; Department of Gastroenterology, Cambridge University Hospital NHS Foundation Trust, Cambridge, United Kingdom; Department of Medicine, University of Cambridge, Cambridge, United Kingdom; Department of Gastroenterology, Royal Devon University Healthcare NHS Foundation Trust, Exeter, United Kingdom; IBD Research Group, University of Exeter, Exeter, United Kingdom; Department of Gastroenterology, St Marks Hospital and Academic Institute, Gastroenterology, London, United Kingdom; Department of Metabolism, Digestion and Reproduction, Imperial College London, London, United Kingdom; Department of Gastroenterology, Cambridge University Hospital NHS Foundation Trust, Cambridge, United Kingdom; Department of Medicine, University of Cambridge, Cambridge, United Kingdom; Department of Gastroenterology, Royal Devon University Healthcare NHS Foundation Trust, Exeter, United Kingdom; IBD Research Group, University of Exeter, Exeter, United Kingdom

**Keywords:** anti-TNF, loss of response, pharmacogenetics, HLA-DQA1*05:01, HLA-DQA1*05:05

## Abstract

**Background and Aims:**

Loss of response (LoR) is a major limitation of anti-tumor necrosis factor (anti-TNF) therapy in inflammatory bowel disease (IBD). It can result from immunogenicity or other, less well-defined mechanisms. Specific HLA alleles have been linked to immunogenicity, but their association with LoR are not fully understood. In this study, we aimed to assess the relationship between HLA alleles and LoR, and investigate the impact of concomitant immunomodulator use.

**Methods:**

LoR and sustained response to infliximab or adalimumab were defined in 25 642 IBD patients from the IBD BioResource. We applied multivariable Cox proportional hazards models to assess the effect of HLA alleles on time to LoR. The effect of concomitant immunomodulator use was also evaluated. Significantly associated alleles were further tested in patients treated with ustekinumab and vedolizumab.

**Results:**

HLA-DQA1*05:01 was associated with reduced time to LoR in infliximab-treated patients (*P *= 5.34E-07), while HLA-DQA1*05:05 was associated with reduced time to LoR in adalimumab-treated patients (*P *= 3.20E-05). Neither allele was associated with LoR to ustekinumab or vedolizumab. Concomitant use of immunomodulators conferred a protective effect against LoR to infliximab and adalimumab in carriers of HLA-DQA1*05:01 and HLA-DQA1*05:05, respectively. However, this protective effect was not observed in adalimumab-treated patients who carried neither allele subtype (*P *= .11).

**Conclusions:**

Our findings highlight distinct associations between HLA-DQA1*05 allele subtypes and time to LoR of infliximab and adalimumab in IBD-treated patients. The protective effect of immunomodulator use is allele-specific for adalimumab. These results provide a rationale for incorporating HLA testing into personalized anti-TNF management to optimize treatment durability.

## 1. Introduction

Immunogenicity, the development of anti-idiotypic antibodies to adalimumab and infliximab, often results in low drug levels and treatment failure of anti-tumor necrosis factor (anti-TNF) drugs.^1^ Carriage of the HLA class II allele group HLA-DQA1*05 is associated with an increased risk of immunogenicity to infliximab and adalimumab in patients with inflammatory bowel disease (IBD).^2^^,^^3^ Recent studies using four-digit HLA resolution have further refined this association: HLA-DQA1*05:01 was linked to immunogenicity to infliximab, while HLA-DQA1*05:05, which encodes an identical α chain but is bound to a different β chain in the heterodimeric cell surface receptor, was associated with immunogenicity to adalimumab.^4^^,^^5^ While these studies focused on immunogenicity as their primary outcome, this is clinically less relevant than loss of response (LoR). Not all detectable anti-drug antibodies result in drug clearance or LoR; conversely, not all LoR is a consequence of immunogenicity. Furthermore, the follow-up periods for previous studies were relatively short. It is unclear whether HLA alleles previously associated with immunogenicity are also associated with reduced time to LoR to infliximab and adalimumab and whether concomitant immunomodulator use can modulate this risk.

In this study, we conducted a retrospective cohort study to evaluate the association between four-digit HLA alleles imputed at seven genes (HLA-A, HLA-B, HLA-C, HLA-DRB1, HLA-DQA1, HLA-DQB1, HLA-DPB1) and time to LoR to anti-TNF treatment. We modeled whether pretreatment pharmacogenetic testing could identify individuals at increased risk of LoR, and therefore individualize the choice of anti-TNF therapy and the need for combination immunomodulator therapy, or use of an alternative class of drug.

## 2. Methods

Patients treated with infliximab and adalimumab were identified from the UK National Institute for Health and Care Research (NIHR) IBD BioResource^6^ (IBDBR), a panel of recallable patients with IBD recruited from more than 100 hospitals across the UK between 2017 and 2024. Baseline phenotypic and drug response data were collected by case note review at enrollment and updated by research sites between January 2023 and August 2025. Genotyping was undertaken with the UKBiobank ThermoFisher genotyping array and HLA alleles were imputed from single nucleotide polymorphism data^7^ (detailed methods are given in the Supplementary Methods).

Patients of European ancestry who had an initial response to anti-TNF therapy assessed between weeks 6 and 20 of treatment were eligible for inclusion. The primary outcome was subsequent LoR defined as symptomatic activity that warranted treatment cessation and switch to an alternative drug or surgery. These decisions were made by clinical teams based on symptoms, biomarkers, and/or endoscopic assessment. Sustained response (SR) was defined as persistence on treatment with no LoR, or elective withdrawal due to sustained remission or pregnancy (Supplementary Methods). Patients also recruited to the PANTS cohort^1^ were excluded from this analysis. Time to LoR was recorded in days. Patients flaring on treatment whose remission was recaptured allowing continued use of index treatment (eg, by escalation of biologic dose, use of a course of corticosteroids or immunosuppressant introduction, or dose escalation) were defined as responders. Concomitant immunomodulator use was defined as use of azathioprine, mercaptopurine, or methotrexate before or within 4 weeks of initiating anti-TNF therapy. Exploratory analyses were also undertaken in patients treated with ustekinumab and vedolizumab.

Assuming an additive genetic model, we conducted an association study between HLA alleles and time to LoR using a multivariable Cox-proportional hazards regression (R package “survival” v.3.5-8^8^^,^^9^) with the following covariates: gender, disease type, drug start year, drug order, concomitant use of immunomodulator, and four genotype-derived principal components. Heterogeneity of effect sizes was assessed using Cochran’s Q test.^10^ Significance thresholds for HLA association and heterogeneity testing were defined using Bonferroni’s correction as 2.5E-04 and 1.25E-02, respectively. A significance level of 0.05 was applied to all other statistical analyses. All analyses were conducted in R v.4.3.1.

## 3. Results

The IBDBR comprised 25 642 patients with up-to-date drug response and genotyping data at the time of analysis. After quality control (Methods) we identified 779, 744, 337, and 186 patients with LoR to infliximab, adalimumab, vedolizumab, and ustekinumab and 1693, 1512, 1082, and 852 patients with SR respectively. The demographic and clinical characteristics of these patients are shown in Table 1.

**Table 1.** Phenotype distribution of patients used in the analysis.

	Infliximab (*N* = 2472)		Adalimumab (*N* = 2256)		Vedolizumab (*N* = 1419)		Ustekinumab (*N* = 1038)	
Drug response	LoR	SR	LoR	SR	LoR	SR	LoR	SR
	779	1693	744	1512	337	1082	186	852
**Sex**								
** Male**	407	873	348	794	183	532	99	380
** Female**	372	820	396	718	154	550	87	472
**Diagnosis**								
** CD**	513	1092	563	1153	163	416	157	674
** UC**	248	565	166	323	171	634	28	167
** IBDU**	18	36	15	36	3	32	1	11
**Age at diagnosis (median)**	25	27	25	27	28	32	23	27
**Smoking at diagnosis**								
**Current smoker**	200	371	200	371	69	214	48	248
**Ex smoker**	135	277	121	257	68	291	20	140
**Never smoker**	366	866	362	740	157	469	96	382
**Missing**	78	179	61	144	43	108	22	82
**Drug prescription order**								
** 1st**	659	1452	522	1120	99	455	17	97
** 2nd**	106	199	210	360	135	417	72	396
** 3rd**	11	39	11	27	82	167	76	284
** 4th**	3	3	1	5	21	43	21	75
**Year of drug start (median)**	2016	2018	2017	2019	2019	2020	2020	2021
**Disease duration (years)**	5	4	6	7	7	10	11	12
**Concomitant use of immunomodulator**								
** Thiopurine ** (azathioprine or mercaptopurine)	310	905	222	510	66	181	33	110
** Methotrexate**	42	63	35	57	10	19	5	22
** Other**	5	18	10	5	5	21	2	4
** None**	256	376	337	637	202	646	126	560
** Missing**	166	331	140	303	54	215	20	156
**Immunomodulator treatment duration**								
** <3 months**	24	59	24	37	2	14	3	13
** 3-12 months**	60	91	33	69	8	50	4	19
** >12 months**	272	835	210	466	71	156	33	103
** Missing**	1	1	0	0	0	1	0	1
**Montreal location classification (CD only)**								
** L1**	156	316	212	443	62	134	53	241
** L2**	124	325	109	280	36	116	33	151
** L3**	214	399	218	366	57	143	65	253
** Upper GI (L4)**	50	92	42	89	18	25	20	44
** Missing**	19	52	24	64	8	23	6	29
**Montreal behavior classification (CD only)**								
** B1**	281	671	284	673	81	255	79	349
** B2**	125	222	169	282	52	97	43	185
** B3**	78	127	74	122	17	33	26	83
** Missing**	29	72	36	76	13	31	9	57
**Perianal disease (CD only)**								
** Yes**	213	447	192	310	49	116	63	223
** No**	265	586	332	775	101	275	86	407
** Missing**	35	59	39	68	13	25	8	44
**Montreal extent classification (UC/IBDU only)**								
** E1**	22	43	20	42	10	67	3	17
** E2**	111	291	80	167	80	318	12	82
** E3**	101	179	64	113	67	208	11	60
** Missing**	32	88	17	37	17	73	3	19

CD, Crohn’s disease, UC, ulcerative colitis; IBDU, inflammatory bowel disease undetermined; GI, gastrointestinal.

**Figure 1.**
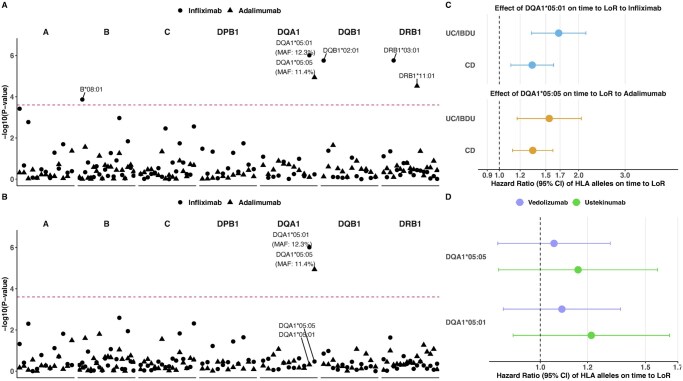
(A) *P*-values for the association between HLA alleles and time to loss of response to infliximab and adalimumab. The MAF of DQA1*05:01 and DQA1*05:05 were shown in the figure. (B) *P*-values for the association between HLA alleles and time to loss of response to infliximab and adalimumab, after conditioning on the most significant allele for each drug (DQA1*05:01 for infliximab and DQA1*05:05 for adalimumab). (C) Effects of DQA1*05:01 and DQA1*05:05 on time to loss of response in patients with different disease types. (D) Effects of DQA1*05:01 and DQA1*05:05 on time to loss of response of vedolizumab and ustekinumab. LoR, loss of response; UC, ulcerative colitis; IBDU, inflammatory bowel disease unclassified; CD, Crohn’s disease; MAF, minor allele frequency; CI, confidence interval.

Among all tested alleles, HLA-DQA1*05:01 (hazard ratio [HR], 1.46 [95% CI: 1.26-1.69], *P *= 5.34E-07) and HLA-DQA1*05:05 (HR, 1.37 [95% CI: 1.18-1.59], *P *= 3.20E-05) were the most significantly associated with time to LoR to infliximab or adalimumab, respectively (Figure 1A). No additional alleles reached significance after conditioning on these two alleles (Figure 1B). In both analyses, there was no evidence of heterogeneity of effect by disease type (Figure 1C). Neither HLA-DQA1*05:01 nor HLA-DQA1*05:05 was associated with LoR to vedolizumab or ustekinumab (Figure 1D).

**Figure 2.**
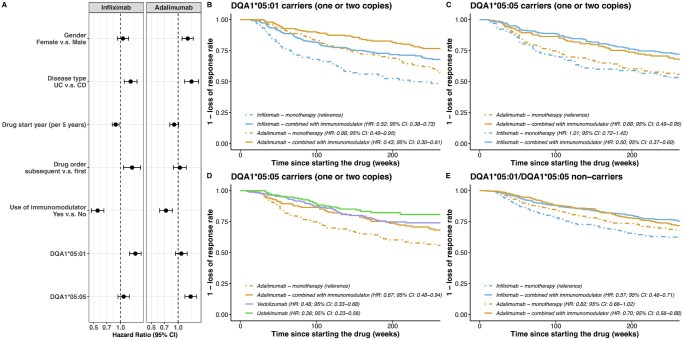
(A) Effects of different factors on time to loss of response from the multivariable model. (B) Kaplan–Meier curves showing the time to loss of response to adalimumab and infliximab in HLA-DQA1*05:01 carriers (one or two copies), stratified by immunomodulator use. (C) Kaplan–Meier curves showing the time to loss of response to adalimumab and infliximab in HLA-DQA1*05:05 carriers (one or two copies), stratified by immunomodulator use. (D) Kaplan–Meier curves showing the time to loss of response to adalimumab, vedolizumab, and ustekinumab in HLA-DQA1*05:05 carriers (one or two copies). (E) Kaplan–Meier curves showing the time to loss of response to adalimumab and infliximab in HLA-DQA1*05:01/HLA-DQA1*05:01 non-carriers, stratified by immunomodulator use. The *x*-axis was truncated at 250 weeks due to the low number of observations after 250 weeks. UC, ulcerative colitis; CD, Crohn’s disease; CI, confidence interval; HR: hazard ratio.

In addition to HLA-DQA1*05:01 carriage, treatment for ulcerative colitis (UC), use as a subsequent (second line) biologic, and earlier era of treatment initiation were all independently associated with a shorter time to LoR to infliximab. For adalimumab, as well as HLA-DQA1*05:05 carriage, female sex and treatment for UC were independently associated with a shorter time to LoR. Overall immunomodulator use was protective against LoR for both drugs (Figure 2A), with different magnitudes of benefit according to genotype (see below). Sensitivity analyses showed that concomitant methotrexate was associated with a shorter time to LoR to infliximab than concomitant thiopurines (HR, 1.50 [95% CI: 1.08-2.08], *P *= 1.57E-02). In contrast, no difference was observed in time to LoR in patients treated with adalimumab and concomitant methotrexate or a thiopurine (HR, 1.32 [95% CI: 0.92-1.90], *P *= 1.30E-01).

Overall, 22.5% and 20.7% of patients in the cohort carried one or more HLA-DQA1*05:01 or HLA-DQA1*05:05 alleles, respectively.

In patients who carried at least one HLA-DQA1*05:01 allele, infliximab monotherapy was associated with the shortest time to LoR. Time to LoR was similar in patients treated with infliximab in combination with an immunomodulator and adalimumab monotherapy and longest in those treated with adalimumab combination therapy (HR, 0.60 [95% CI: 0.43-0.84], *P *= 2.82E-03 for the comparison between adalimumab combination therapy and adalimumab monotherapy) (Figure 2B, Table 2). However, the benefits of a concomitant immunomodulator alongside adalimumab in HLA-DQA1*05:01 carriers was modest: the number needed to treat (NNT) with an immunomodulator to prevent one event of LoR within 1 year of adalimumab initiation was 65.

**Table 2.** The effect (hazard ratio) of concomitant immunomodulator use (vs monotherapy) on time to loss of response among patients with different HLA alleles.

Drug	Allele carriage	HR (95% CI)	*P*-value
**Infliximab**	Carriers of HLA-DQA1*05:01	0.55 (0.40-0.77)	4.34E-04
	Carriers of HLA-DQA1*05:05	0.50 (0.36-0.71)	7.26E-05
	Non-carriers of HLA-DQA1*05:01/05:05	0.58 (0.47-0.71)	4.22E-07
**Adalimumab**	Carriers of HLA-DQA1*05:01	0.60 (0.43-0.84)	2.82E-03
	Carriers of HLA-DQA1*05:05	0.67 (0.48-0.93)	1.83E-02
	Non-carriers of HLA-DQA1*05:01/05:05	0.84 (0.68-1.04)	1.10E-01

In patients who carried at least one HLA-DQA1*05:05 allele, infliximab combination therapy, adalimumab combination therapy, vedolizumab, and ustekinumab were all associated with a longer time to LoR compared to adalimumab monotherapy (Figure 2C and D).

In patients who carried neither HLA-DQA1*05:01 nor HLA-DQA1*05:05, there was a protective effect of immunomodulators on time to LoR in infliximab-treated patients (Figure 2E). In contrast, among adalimumab-treated patients there was no significant benefit of combination therapy on time to LoR (HR, 0.84 [95% CI: 0.68-1.04], *P *= 0.11).

## 4. Discussion

This study presents the largest analysis to date linking HLA alleles with time to LoR to anti-TNF drugs in patients with IBD. We have demonstrated that HLA-DQA1*05:01 is associated with a shorter time to LoR in infliximab-treated patients whilst HLA-DQA1*05:05 is associated with a shorter time to LoR in adalimumab-treated patients. These associations were observed in patients with Crohn’s disease and ulcerative colitis, but neither allele was associated with LoR to vedolizumab or ustekinumab.

Immunogenicity is a key contributor to LoR, but the presence of anti-drug antibodies is neither sufficient nor necessary for its occurrence. Our results extend previously reported four-digit HLA allele subtype associations with anti-TNF immunogenicity^4^^,^^5^ to show an association with treatment LoR. This was possible because our study was conducted in a large cohort of rigorously characterized patients with considerably longer follow-up than previous studies. Together, these studies show the importance of employing four-digit HLA resolution to identify drug-specific associations with HLA-DQA1*05 allele subtypes.

**Figure 3.**
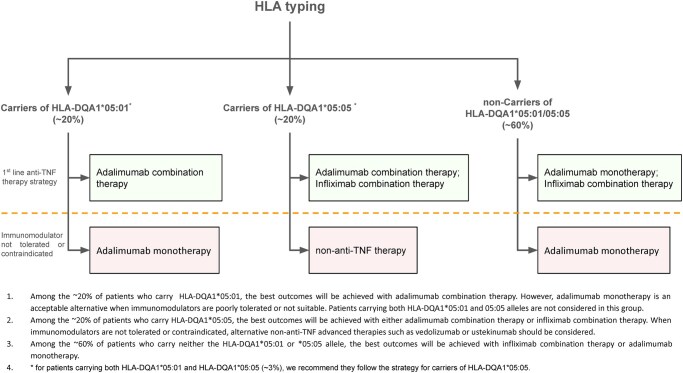
Recommended anti-tumor necrosis factor (anti-TNF) treatment strategy. TNF, tumour necrosis factor.

Concomitant use of immunomodulators was associated with a reduced rate of LoR in infliximab and genetically defined subsets of adalimumab-treated patients. The protective effect on LoR to adalimumab was only seen in patients who carried either HLA-DQA1*05:01 (where the benefit of immunomodulators was modest) or HLA-DQA1*05:05, and not in individuals who did not carry either suballele. This suggests that HLA testing could usefully guide treatment decisions regarding concomitant immunomodulator usage alongside adalimumab therapy. Based on these findings, we propose the personalized treatment strategy outlined in Figure 3.

We acknowledge several important limitations of this study. First, as a consequence of the retrospective study design, patients in whom treatment outcomes could not be confidently assigned were excluded, reducing the sample size and introducing selection bias. Second, consistent with clinical practice, LoR was defined clinically rather than by endoscopic assessment. Third, we lacked anti-TNF drug levels to identify if LoR was mediated through drug clearing antibodies, and we did not distinguish between intravenous and subcutaneous formulations of infliximab. Fourth, data relating to immunomodulator dose or blood levels were not available. Finally, our association study was limited to patients with IBD of European descent.

In summary, we demonstrate that HLA-DQA1*05:01 is associated with reduced time to LoR in infliximab-treated patients, while HLA-DQA1*05:05 is associated with reduced time to LoR in adalimumab-treated patients. Concomitant use of immunomodulators was linked to a reduced time to LoR in both treatment groups, but this effect was not observed for adalimumab in the 60% of individuals who lack either HLA-DQA1*05:01 or *05:05 suballeles. These findings suggest that HLA testing may help guide the selection of anti-TNF agents, use of concomitant immunomodulators, and identification of individuals who might benefit from increased, proactive therapeutic drug monitoring to mitigate the risk of LoR to anti-TNF therapy.

## Data Availability

The data underlying this article will be shared at aggregate/population level on reasonable request to the corresponding author. Patient-level data underlying this study are available to researchers subject to the access processes of the UK IBD BioResource, detailed further at https://www.ibdbioresource.nihr.ac.uk/index.php/resources/.
